# Comprehensive Review on Distal Femur Fractures: From Epidemiology to Treatment Strategies

**DOI:** 10.7759/cureus.57937

**Published:** 2024-04-09

**Authors:** Rahul Singh, Ratnakar Ambade, Suhas Landge, Saksham Goyal, Sachin Goel

**Affiliations:** 1 Department of Orthopaedics, Jawaharlal Nehru Medical College, Datta Meghe Institute of Higher Education and Research, Wardha, IND; 2 Department of Orthopaedic Surgery, Jawaharlal Nehru Medical College, Datta Meghe Institute of Higher Education and Research, Wardha, IND

**Keywords:** clinical implications, classification, anatomy, treatment strategies, epidemiology, distal femur fractures

## Abstract

Distal femur fractures present a substantial orthopedic challenge, necessitating a comprehensive exploration spanning epidemiology, anatomy, classification, diagnosis, and treatment strategies. This review thoroughly analyzes the multifaceted aspects surrounding distal femur fractures. It delves into the definition and epidemiology, shedding light on the incidence, age distribution, and associated risk factors. An exhaustive examination of the distal femur's anatomy, encompassing ligaments and tendons, establishes the groundwork for understanding fracture patterns and subsequent classification according to the AO Foundation/Orthopaedic Trauma Association (AO/OTA) system. Diagnostic considerations encompass physical examination and various imaging modalities, emphasizing the critical importance of prompt and accurate assessment. The extensive discussion on treatment options ranges from non-surgical management, including casting and traction, to surgical interventions, such as open reduction and internal fixation, intramedullary nailing, and external fixation. The implications for clinical practice underscore the necessity for tailored approaches based on fracture characteristics to optimize patient outcomes. However, this review also emphasizes areas necessitating further investigation, including exploring predictive biomarkers, advanced surgical techniques, and innovative rehabilitation protocols. Insights from long-term outcomes and quality-of-life assessments in diverse populations offer promising avenues for enhancing the comprehensive management of distal femur fractures. Continuous research in these areas can refine treatment strategies and elevate the standard of care for individuals grappling with this intricate orthopedic condition.

## Introduction and background

Distal femur fractures in the lower part of the femur near the knee joint involve a break in the bone's distal portion, encompassing the metaphysis and epiphysis. These fractures range from simple to complex intra-articular fractures [1]. Despite their relatively infrequent occurrence, they carry the potential for significant morbidity and functional impairment, particularly concerning the knee joint. Hence, understanding their epidemiology, causes, and treatment options is imperative for healthcare professionals and researchers to enhance patient outcomes and refine healthcare practices [2].

A thorough grasp of the distal femur's anatomy is essential to understand distal femur fractures comprehensively. Serving as a crucial weight-bearing structure essential for knee joint stability and mobility, the distal femur comprises the metaphysis, epiphysis, and an array of ligaments and tendons contributing to knee integrity [3]. This review endeavors to delve deeply into distal femur fractures, encompassing pivotal aspects, such as epidemiology, anatomy, classification, diagnosis, treatment options, complications, and potential future research avenues. By consolidating existing knowledge and identifying knowledge gaps, this review aims to propel clinical practices forward and elevate patient care standards for individuals grappling with distal femur fractures.

## Review

Epidemiology of distal femur fractures

Incidence Rates in Different Populations

The occurrence of distal femur fractures varies across different populations [4]. For instance, in developed countries, the incidence rate falls within the range of 4.7 to 8.7 fractures per 100,000 patients [4,5]. These fractures exhibit a bimodal distribution, with a notable peak observed in young adults, predominantly males, and a significant surge in incidence after age 60, particularly among females [4]. Most of these fractures result from low-energy injuries, notably falls from standing height [4]. Studies indicate a six-month mortality rate of 16% for distal femur fractures, escalating to 30% within one year, with approximately half of the fractures occurring in patients over 70 years old [6]. The incidence rate is a metric to gauge the frequency at which new instances of a condition emerge over a specified period among a population at risk of the event [7].

Age and Sex Distribution

Distal femur fractures exhibit a bimodal distribution, characterized by a peak occurrence in young adults, primarily males, which diminishes until age 50 and is often associated with high-energy polytrauma. Subsequently, after age 60, there is a marked surge in the incidence of distal femur fractures across both sexes, with a significant female predominance [1,8,9]. The average age at the time of fracture stands at 62.2 years, with males averaging 44.0 years and females 71.6 years [8]. Low-energy injuries constitute the most prevalent mode of injury in both sexes, accounting for 97% of cases, with approximately 61% attributed to falls from standing height [8]. Notably, there has been a shift in the sex ratio, with women now comprising the majority (one man to two women), and the population is progressively older, with an average age of 61 years at the time of fracture [1].

Common Causes and Risk Factors

**Figure 1:**
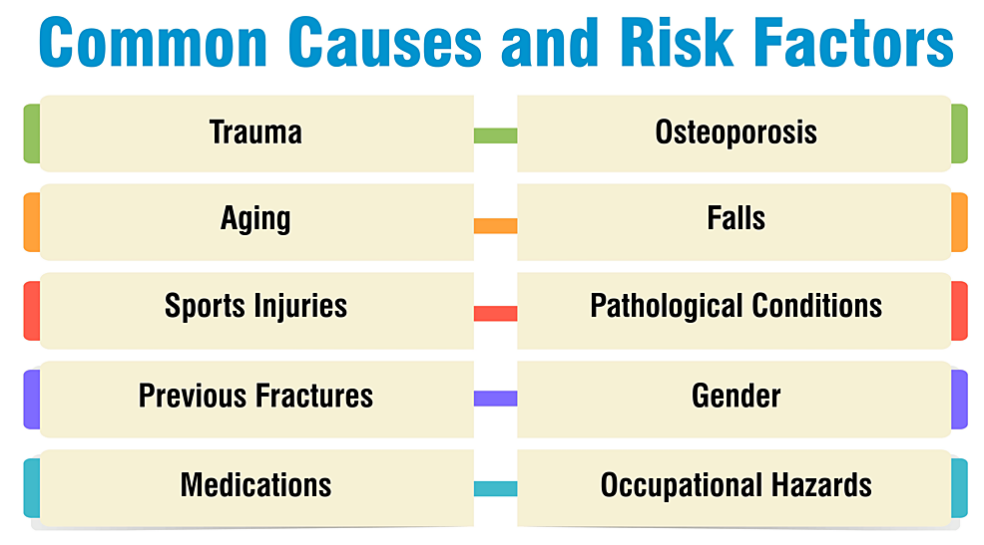
Common causes and risk factors Image credit: Dr. Rahul Singh

Distal femur fractures can arise from differing mechanisms depending on the age group. High-energy trauma, such as motor vehicle accidents or falls from significant heights, is a common cause, particularly among young adults. Conversely, in the elderly population, these fractures often occur due to low-energy injuries, with more than 85% of cases attributed to such incidents, commonly stemming from falls from standing height [8]. Remarkably, around 61% of distal femur fractures in older individuals result from falls from a standing position. This dichotomy in causative factors contributes to the bimodal distribution of these fractures, with a peak incidence in young adults, often males, due to high-energy trauma and a subsequent rapid rise in frequency after the age of 60, especially among women, owing to low-energy injuries typically associated with osteoporosis. Common symptoms accompanying distal femur fractures include pain upon weight-bearing, swelling, bruising, tenderness to touch, and potential deformity [8,10]. Common causes and risk factors are shown in Figure 1.

Associated Injuries and Comorbidities

Distal femur fractures often coincide with additional injuries and underlying health conditions. In elderly patients who frequently sustain distal femur or hip fractures, compromised bone quality and the presence of multiple medical comorbidities contribute to a heightened mortality risk [11,12]. Conversely, in young patients subjected to high-energy trauma, fractures tend to be intra-articular, characterized by increased displacement and comminution, and are often accompanied by more severe soft tissue compromise. The primary systemic concern in these cases is multiorgan injury (polytrauma), followed by other concomitant orthopedic injuries [13]. Low-energy injuries, such as falls from standing height, are the predominant mode of injury across both sexes, accounting for approximately 61% of cases [11]. While a study noted no significant correlation between comorbidities and non-union rates of distal femur fractures, it identified several predictive factors [11].

Classification of distal femur fractures

AO/OTA Classification System

The AO Foundation/Orthopaedic Trauma Association (AO/OTA) classification system, originating from the Müller classification and updated in 2007, places significant importance on evaluating fracture severity, focusing specifically on fragment integrity, displacement, impaction, and dislocation. It categorizes fractures into three main types (A, B, and C), further delineating them based on the extent of fragmentation and displacement. A subsequent refinement in 2018 aimed to streamline the system, making it more precise, concise, and clinically relevant for adults and children. This revision addressed the need for improved terminology, typography, and the frequency and applicability of codes [14,15]. Known for its reliability, reproducibility, and utilization of anatomically precise terms, the OTA Classification is easily grasped and utilized in clinical practice [16].

Intra-articular Versus Extra-articular Fractures

Intra-articular fractures involving the joint space are deemed more critical than extra-articular fractures due to their potential to cause complications, such as articular cartilage damage and subsequent joint issues. Addressing these injuries may necessitate arthroscopic debridement, chondroplasty, or joint replacement to rectify associated problems [17]. Conversely, extra-articular fractures do not breach the joint and are generally less severe, posing a lower risk of joint-related complications [18]. The treatment approach and long-term functional outcomes diverge between intra-articular and extra-articular fractures. For instance, extra-articular fractures in hand fractures may exhibit superior total active motion and grip strength compared to intra-articular fractures, although statistical significance may not always be reached [19]. Moreover, the management of these fractures differs, with surgical intervention often required for intra-articular fractures, while certain extra-articular fractures may be amenable to conservative management [20].

Clinical presentation and diagnosis

Signs and Symptoms of Distal Femur Fractures

**Figure 2:**
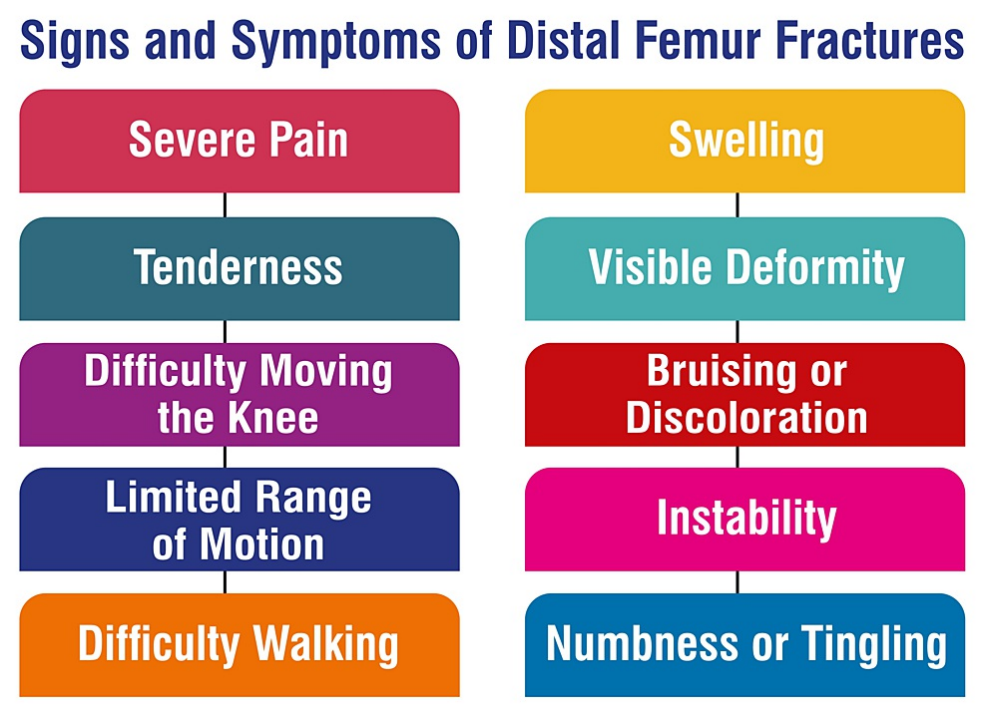
Signs and symptoms of distal femur fractures Image credit: Dr. Rahul Singh

Signs and symptoms associated with distal femur fractures encompass pain upon weight-bearing, swelling, bruising, tenderness upon palpation over the thigh or knee, and potential deformity [10,21,22]. Patients may also be unable to bear weight, and the affected leg may appear shorter than the unaffected one [22]. These fractures typically occur following twisting motions or falls, with patients frequently seeking medical attention after experiencing a fall or traumatic incident [10,12]. Diagnosis primarily relies on radiographic imaging, with computed tomography (CT) studies often necessary to evaluate potential intra-articular extension [10,21,22]. The signs and symptoms of distal femur fractures are shown in Figure 2.

Physical Examination and Imaging Modalities (X-ray, CT, and MRI)

X-rays are the initial imaging modality for suspected distal femur fractures due to their widespread availability and ability to provide detailed images of bone structures. X-rays offer crucial information regarding the fracture's presence, location, and characteristics, including displacement and potential involvement of the joint space. They are instrumental in quickly confirming the diagnosis and guiding initial treatment decisions [23]. CT scans are pivotal in further assessing complex or intra-articular distal femur fractures. CT scans provide valuable insights into the fracture's extent, intra-articular involvement, and associated soft tissue damage by offering detailed cross-sectional images of the affected area. This information benefits surgical planning, enabling orthopedic surgeons to precisely evaluate the fracture pattern and determine the most appropriate intervention [23,24]. Although not always the primary imaging modality for acute fractures, magnetic resonance imaging (MRI) can offer unique advantages in some distal femur fractures. MRI excels in evaluating soft tissue structures, including ligaments, tendons, and cartilage, which may be affected in conjunction with the fracture. In addition, MRI is valuable for detecting subtle or stress fractures that may not be readily apparent on conventional X-rays or CT scans. While MRI may not be routinely performed for all distal femur fractures, its use can be beneficial in specific clinical scenarios where soft tissue involvement or complex fracture patterns are suspected [23-25].

Importance of Prompt and Accurate Diagnosis

The prompt and precise diagnosis of distal femur fractures holds paramount importance due to the potential for associated morbidity and mortality. A comprehensive approach involving a detailed history, thorough physical examination, and appropriate diagnostic imaging is essential for achieving an accurate diagnosis. The history should encompass details of the traumatic event, onset and location of pain, and any prodromal symptoms, particularly for fractures occurring with overuse or minimal trauma [26]. The physical examination entails assessing for deformity, instability, and crepitation, with a thorough head-to-toe examination crucial to identifying any associated injuries [26]. Swift and accurate diagnosis is essential for timely and appropriate treatment, often surgical, to minimize complications and optimize patient outcomes [27].

Treatment options

Non-surgical Management

Indications and considerations: The distal femur, a weight-bearing joint prone to fractures, typically necessitates surgical intervention to stabilize fractures due to its anatomy. Commonly utilized procedures include locking plate osteosynthesis, retrograde intramedullary (IM) nail osteosynthesis, and screw fixation [27]. Non-surgical treatment is rarely considered and is usually reserved for patients ineligible for surgery [1]. Current management options for distal femur fractures include distal femur replacement (DFR), surgical fixation (SF), or conservative management, with surgical treatment typically indicated for most displaced and intra-articular fractures [28]. Treatment goals aim to restore length, alignment, rotation, and articular congruence while mitigating these fractures' high morbidity and mortality [12].

Casting and traction techniques: Distal femur fractures are predominantly managed surgically, with non-surgical methods, such as casting and traction, being less common. However, in pediatric femur fractures, there is a discussion regarding using 90-90 traction followed by delayed spica casting as a treatment option [29]. In addition, skeletal traction followed by spica casting has been considered for femur fractures in general [30]. While non-surgical approaches like casting and traction are mentioned as potential treatments, it is imperative to acknowledge that surgical intervention is typically the primary approach for distal femur fractures. Casting and traction are less frequently employed and may be reserved for specific cases, such as in pediatric patients or when surgical intervention is not feasible [31].

Surgical Management

Open reduction and internal fixation (ORIF): ORIF is a surgical procedure employed to repair fractured bones by utilizing screws, plates, or an IM rod to stabilize the bone [32-36]. The selection of fixation devices is contingent upon factors such as the type and location of the fracture, as well as the surgeon's preference and expertise. Screws and plates are typically employed for fractures near the joint surface, while IM nails are favored for fractures farther from the joint [33,35,36]. The surgical approach for ORIF is determined by the fracture's location and type, with anterolateral, medial, and lateral approaches being the most common [33,35,36]. The anterolateral approach is typically utilized for lateral distal femur fractures, whereas the medial approach is preferred for fractures on the medial side. The choice of approach is influenced by the surgeon's experience and preference, alongside the fracture's location and type.

IM nailing: IM nailing is a surgical procedure commonly employed to treat femoral shaft fractures in adults, involving the insertion of a metal rod into the bone's medullary cavity. This technique maintains the fracture site's anatomical structure and creates a conducive environment for fracture healing [37,38]. Widely regarded as the "gold standard" for femoral shaft fracture treatment, IM nailing offers advantages, such as a shorter hospital stay, rapid fracture union, and early limb function restoration [38]. Various types of IM nails and associated surgical techniques are available, with procedures performed through antegrade (proximal to distal) or retrograde (distal to proximal) nailing approaches [39,40]. IM nailing yields a stable fixation construct with high union rates and low complication rates when executed meticulously [40]. The selection of nailing techniques, including reamed versus unreamed and locked versus unlocked nailing, may depend on factors, such as fracture type, patient characteristics, and concurrent injuries [38,40].

External fixation: External fixation constitutes a surgical intervention wherein metal pins or screws are inserted into the bone through small incisions in the skin and muscle. These pins and screws are then connected to an external bar to maintain the proper alignment and position of fractured bones [41]. The primary objective of external fixation is to preserve the fracture's length, alignment, and rotation [42]. It can be employed either as a temporary measure or as a definitive treatment, and in some cases, it may be combined with partial internal fixation if deemed necessary [42]. External fixation is joint in pediatric cases and situations where the skin over the fracture site has sustained damage [43]. Orthopedic surgeons at trauma centers must be proficient in the techniques and equipment utilized for external fixation [42].

Total knee arthroplasty (TKA) in select cases: TKA is a surgical procedure utilized to treat severe knee arthritis, involving the replacement of the affected knee joint with artificial components [44]. Typically recommended when conservative measures such as medication and physical therapy fail to provide sufficient relief from pain and disability, TKA has demonstrated success in various case studies and reports. For instance, a 72-year-old female suffering from severe tricompartmental osteoarthritis experienced significant mobility improvement and pain reduction following a successful right TKA [45]. In addition, TKA has been proposed as an effective treatment for end-stage ochronosis arthropathy, supported by case reports and systematic reviews [46]. The widespread adoption of TKA is evidenced by high case volumes reported in the United States, with an estimated 526,000 to 538,000 procedures performed in 2020 alone [47].

Complications and outcomes

Common Complications Associated With Distal Femur Fractures

Typical complications linked with distal femur fractures encompass pin-tract infection, deep infection, loss of reduction, malunion, and knee stiffness [12,22,48]. These fractures commonly exhibit comminution, intra-articular involvement, periprosthetic association, and soft tissue damage, rendering their management notably challenging [1,48]. Preserving soft tissue integrity is pivotal for fostering fracture healing and can significantly influence the outcomes of distal femur fractures [1]. Despite advancements in fixation techniques, distal femoral fractures can lead to persistent disability and inferior clinical outcomes, underscoring the imperative for comprehensive management strategies [1].

Impact on Joint Function and Mobility

Distal femur fractures can profoundly affect joint function and mobility. Post-surgical physical rehabilitation aims to optimize functional outcomes, restore joint mobility, and bolster muscular strength [49]. The locking plate system, a surgical technique, facilitates early mobility, rapid functional recovery, and favorable radiological results with minimal morbidity [1]. However, despite advancements in fixation techniques, distal femoral fractures often result in persistent disability and compromised clinical outcomes. Preserving soft tissue integrity is paramount for fracture healing, and the prognosis of distal femoral fractures hinges on bony reconstruction and effective soft tissue management [28]. Treatment decisions should be based on a comprehensive assessment of the fracture, the patient's condition, and each approach's potential risks and benefits to optimize functional outcomes and restore joint mobility.

Long-Term Outcomes and Quality of Life

Long-term outcomes and quality of life following distal femur fractures, particularly among geriatric patients, pose significant concerns. A study has documented the poor functional long-term outcomes among geriatric individuals with distal femoral fractures, emphasizing the heightened risk and the imperative to mitigate medical complications associated with inferior functional outcomes [50]. Another study has underscored the substantial burden of morbidity and mortality within the older adult population stemming from distal femur fractures [48]. The choice of management, whether it involves DFR, SF, or conservative approaches, can profoundly influence the long-term outcomes and quality of life for patients with these fractures [28]. Clinicians must carefully weigh the potential complications and functional outcomes when determining the most suitable treatment approach for distal femur fractures, particularly among the geriatric population.

Rehabilitation and postoperative care

Importance of Early Mobilization

Early mobilization following hip and femur fracture surgery has garnered considerable attention in research, with evidence highlighting its significance in postoperative care. Multiple studies have indicated the advantages of early mobilization, including reduced hospital length of stay, decreased complications, and improved long-term ambulatory function. For instance, a literature review revealed that early ambulation, defined as mobilization within 24 hours after surgery, correlated with lower complication rates and mortality [51]. Another study stressed the importance of early mobilization post-surgical hip procedures, particularly among geriatric patients, to mitigate complications and mortality risks [52]. In addition, initiating physical therapy promptly after geriatric hip fracture surgery has been linked to shorter hospital stays and lower 30-day mortality rates [53]. While the evidence base for early mobilization, especially concerning distal femur fractures, remains somewhat limited, existing studies suggest potential benefits, particularly in facilitating a quicker return to function for geriatric patients [54]. Nevertheless, individual patient factors should be considered, and healthcare professionals' guidance should be followed when determining the appropriate mobilization protocol.

Physical Therapy Protocols

Physical therapy protocols for distal femur fractures typically involve gradually progressing exercises and activities to restore function and strength. According to the Reno Orthopedic Center, most patients with distal femur fractures begin physical therapy at their first postoperative visit. During the initial phase, which lasts approximately six weeks, the focus is on a range of motion exercises and modalities while the bone heals. Around the six-week mark, therapy advances to include stationary bike riding and more aggressive range of motion exercises [55]. As Very Well Health outlines, specific physical therapy exercises recommended for femur fractures include straight leg raises, bridges, clamshells, standing hip extensions, hip abductions, and sit-to-stand exercises [56]. Moreover, an article in Cureus underscores that the primary goal of physical therapy following a distal femur fracture is to restore the patient to baseline function and prevent complications [49]. The rehabilitation aims to enhance the range of motion, strengthen muscles, and facilitate a safe return to weight-bearing activities. The specific protocol may vary based on the individual's condition and the type of fracture, highlighting the importance of following healthcare professionals' guidance to ensure adequate recovery [57].

Monitoring and Managing Complications During Recovery

In the postanesthesia care unit (PACU), monitoring and management of respiratory complications, including airway obstruction and hypoxemia, are essential following general anesthesia [58]. This critical phase demands meticulous observation to address any respiratory issues and ensure optimal patient safety promptly. Rapid intervention is crucial for patients' vulnerability during this immediate postoperative period. Continuous surveillance of vital signs is integral to detecting early signs of complications among postoperative patients [59,60]. Abnormalities in vital signs, such as hypotension and desaturation, can serve as early indicators of impending adverse events. Through continuous monitoring, healthcare providers can swiftly identify deteriorating patients and initiate appropriate interventions to prevent further complications. This proactive approach significantly contributes to patient safety and reduces the risk of adverse outcomes.

The emergence of mobile applications has transformed postoperative care by enabling remote monitoring and support for patients during their transition and recovery phase [61]. These applications facilitate ongoing surveillance, allowing healthcare professionals to identify potential complications and intervene remotely. Leveraging technology in this manner enhances efficiency in postoperative care delivery, leading to improved patient outcomes and more effective early intervention strategies. Adhering to standards for postanesthesia care, thorough evaluation, monitoring, and support are essential throughout a patient's stay in the PACU [62]. In addition, establishing clear criteria for patient discharge to various settings is imperative, with careful consideration given to the roles of physicians and the anesthesia care team in the discharge process. Ensuring a seamless transition from the PACU to subsequent care settings is paramount for optimizing patient safety and maintaining continuity of care.

Future directions and advances

Emerging Technologies in Fracture Fixation

**Figure 3:**
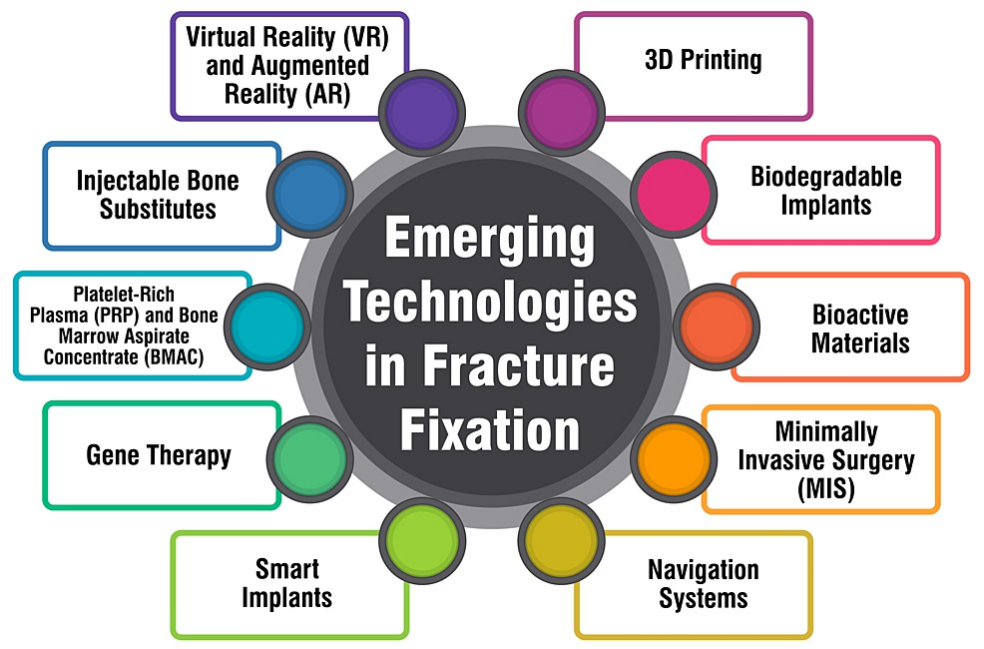
Emerging technologies in fracture fixation Image credit: Dr. Rahul Singh

Cutting-edge developments in fracture fixation encompass innovative technologies, such as computer-assisted preoperative planning, generative design for surgical implants, and advancements in internal fixation materials. A comprehensive review delves into the evolution of a patient-specific finite element analysis (FEA) approach tailored to surgical planning of tibial plateau fractures. This review underscores the effectiveness and feasibility of existing software approaches in facilitating preoperative planning for bone fracture fixation surgery [63]. Moreover, generative design within Fusion 360 software is being explored to engineer novel designs for surgical implants. The focus lies on enhancing the mechanical performance of components while streamlining manufacturing processes and material selections [64]. Furthermore, recent strides in rib fracture fixation entail advancements in internal fixation materials, including the emergence of absorbable alternatives. In addition, there is an exploration into using thoracoscopic surgical stabilization of rib fractures (SSRF) [64]. These advancements signify a transition toward more personalized and innovative approaches to fracture fixation, with a paramount goal of enhancing patient outcomes and ensuring the long-term stability of fracture repairs. Emerging technologies in fracture fixation are shown in Figure 3.

Novel Treatment Modalities and Materials

Generative design for surgical implants: Researchers are harnessing generative design within Fusion 360 software to explore innovative designs for surgical implants. This cutting-edge approach aims to enhance implant components' mechanical performance, streamline manufacturing processes, and optimize material choices. It addresses the limitations associated with traditional surgical implants, which may inadvertently lead to bone mass reduction and an increased risk of future fractures [65]. By leveraging generative design, researchers aim to develop implants that better accommodate the unique biomechanical requirements of each patient, ultimately improving the efficacy and longevity of fracture repair procedures.

Absorbable internal fixation materials: Recent advancements in rib fracture fixation have led to the development of absorbable internal fixation materials, representing a significant area of focus for future research endeavors. These materials are specifically engineered to adapt to the distinct geometric shapes and biomechanical challenges presented by rib fractures. The primary objective is to enhance the convenience and effectiveness of rib fracture fixation by offering solutions that minimize the need for subsequent implant removal procedures [64]. This innovative approach holds promise for revolutionizing the treatment of rib fractures, potentially reducing patient discomfort and complications associated with traditional fixation methods.

Advancements in internal fixation materials for distal femur fractures: Distal femur fractures pose unique challenges in treatment, often requiring complex surgical interventions. Standard procedures include locking plate osteosyntheses, retrograde IM nail osteosyntheses, and screw fixation. In addition, the utilization of advanced internal fixation devices, such as distal femur locking plates and dynamic condylar screws, is contributing to the evolution of treatment modalities for these intricate fractures [27,66]. By incorporating these advanced fixation materials, orthopedic surgeons can better address the complexities of distal femur fractures, ultimately improving patient outcomes and enhancing the stability of fracture repairs.

## Conclusions

This comprehensive review has provided a detailed exploration of the complexities surrounding distal femur fractures, an orthopedic challenge of significant concern. Various dimensions have been scrutinized from epidemiology to treatment strategies to enhance our understanding of this condition. The analysis has highlighted the importance of recognizing fracture patterns and associated risk factors, underscoring the necessity for tailored treatment approaches. These range from conservative measures to surgical interventions, such as open reduction and internal fixation, IM nailing, and external fixation. The implications for clinical practice are profound, offering clinicians valuable insights into optimizing patient outcomes and refining treatment strategies based on individual fracture characteristics. However, as our knowledge evolves, numerous avenues for further investigation emerge. The pursuit of novel biomarkers predicting fracture healing, exploration of advanced surgical techniques, and development of innovative rehabilitation protocols represent promising areas for future research. Moreover, assessing long-term outcomes and quality of life in diverse patient populations can enhance the holistic management of distal femur fractures. Continuous research in these domains promises to refine treatment strategies and ultimately raise the standard of care for individuals grappling with this challenging orthopedic condition.
